# Identification of the sequence determinants of protein *N*-terminal acetylation through a decision tree approach

**DOI:** 10.1186/s12859-017-1699-4

**Published:** 2017-06-02

**Authors:** Kazunori D. Yamada, Satoshi Omori, Hafumi Nishi, Masaru Miyagi

**Affiliations:** 1 Graduate School of Information Sciences, Tohoku University, Sendai, 980-8579 Japan; 2 Artificial Intelligence Research Center, National Institute of Advanced Industrial Science and Technology (AIST), Tokyo, 135-0064 Japan; 3 Center for Proteomics and Bioinformatics, Case Western Reserve University, Cleveland, OH 44106 USA; 4 Department of Nutrition, Case Western Reserve University, Cleveland, OH 44106 USA

**Keywords:** *N*-terminal acetylation, *N*-terminal acetyltransferase, Decision tree, Sequence analysis, Sequence context

## Abstract

**Background:**

*N*-terminal acetylation is one of the most common protein modifications in eukaryotes and occurs co-translationally when the *N*-terminus of the nascent polypeptide is still attached to the ribosome. This modification has been shown to be involved in a wide range of biological phenomena such as protein half-life regulation, protein-protein and protein-membrane interactions, and protein subcellular localization. Thus, accurately predicting which proteins receive an acetyl group based on their protein sequence is expected to facilitate the functional study of this modification. As the occurrence of *N-*terminal acetylation strongly depends on the context of protein sequences, attempts to understand the sequence determinants of *N*-terminal acetylation were conducted initially by simply examining the *N*-terminal sequences of many acetylated and unacetylated proteins and more recently by machine learning approaches. However, a complete understanding of the sequence determinants of this modification remains to be elucidated.

**Results:**

We obtained curated *N-*terminally acetylated and unacetylated sequences from the UniProt database and employed a decision tree algorithm to identify the sequence determinants of *N*-terminal acetylation for proteins whose initiator methionine (^i^Met) residues have been removed. The results suggested that the main determinants of *N*-terminal acetylation are contained within the first five residues following ^i^Met and that the first and second positions are the most important discriminator for the occurrence of this phenomenon. The results also indicated the existence of position-specific preferred and inhibitory residues that determine the occurrence of *N*-terminal acetylation. The developed predictor software, termed NT-AcPredictor, accurately predicted the *N*-terminal acetylation, with an overall performance comparable or superior to those of preceding predictors incorporating machine learning algorithms.

**Conclusion:**

Our machine learning approach based on a decision tree algorithm successfully provided several sequence determinants of *N*-terminal acetylation for proteins lacking ^i^Met, some of which have not previously been described. Although these sequence determinants remain insufficient to comprehensively predict the occurrence of this modification, indicating that further work on this topic is still required, the developed predictor, NT-AcPredictor, can be used to predict *N*-terminal acetylation with an accuracy of more than 80%.

**Electronic supplementary material:**

The online version of this article (doi:10.1186/s12859-017-1699-4) contains supplementary material, which is available to authorized users.

## Background

**Fig. 1:**
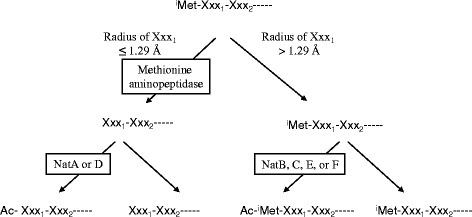
Overview of the major pathways of *N*-terminal processing for eukaryotic proteins. ^i^Met: initiator methionine; Xxx1 and Xxx2: the first and second amino acid residues following ^i^Met, respectively; NatA, B, C, D, E, and F: *N*-terminal acetyltransferases A, B, C, D, E, and F, respectively

*N*-terminal acetylation of proteins (*N* ^α^-acetylation) is a co-translational modification that takes place when the *N*-terminus of the nascent polypeptide is still attached to the ribosome [1]. This modification represents one of the most common protein modifications in eukaryotes, occurring on more than 80% of human proteins [2]. Figure 1 depicts the major pathways of *N*-terminal processing for eukaryotic proteins. The initiator methionine (^i^Met) of the nascent chain is recognized and cleaved off by methionine aminopeptidase if the amino acid residue following the ^i^Met has a radius of gyration not greater than 1.29 Å (i.e., Gly, Ala, Ser, Cys, Thr, Pro, and Val) [3]. Subsequently, *N*-terminal acetylation of the proteins may occur depending on the amino acid sequence context of their *N*-terminal region. Humans possess six *N*-terminal acetyltransferase (Nat) enzymes, which catalyze this reaction (NatA, B, C, D, E, and F). NatA and D act on the nascent chains from which ^i^Met residues have been cleaved off [1]. The substrate specificity of NatD is very strict, and its only known substrates are histone H2A and H4 [4]. Therefore, the majority of acetylation on proteins lacking the ^i^Met residue is catalyzed by NatA. In contrast, NatB, C, E, and F act on nascent chains that retain the ^i^Met residue [1]. Similar to NatA, three of these Nat enzymes, NatB, C, and E constitute ribosomal proteins, whereas NatF is associated with the Golgi surface and specifically acetylates transmembrane proteins [5].

The biological effects of *N*-terminal acetylation had long been unclear because mutant yeast lacking Nat enzymes appeared to grow normally [6]. However, the diverse functions of this modification have begun to be uncovered over the past decade; these include regulations of protein half-life, protein-protein and protein-membrane interactions, subcellular localization, folding, and aggregation [1]. As many proteins are *N*-terminally acetylated, it is expected that new functional roles of this modification will continue to emerge in the future.

*N*-terminally acetylated proteins have been traditionally identified by comparing the *N*-termini of proteins from yeast lacking one or more of Nat enzymes with those expressed in wild-type strains [6–9], and more recently by proteomic approaches [2, 10–13]. These studies identified many acetylated and unacetylated proteins but were unable to determine the complete sequence requirements for this modification, suggesting that the substrate specificity of these enzymes is rather broad [14, 15]. Machine learning approaches have also been utilized for predicting *N*-terminal acetylation based on the amino acid sequence of the *N*-terminal region. The representative methods include NetAcet [16], which exerts simple feed-forward neural networks for prediction, and Motifs tree [17], which utilizes detailed sequence motifs for the input of the decision tree method. These approaches, however, do not provide explicit processing pathways and therefore cannot be used to study sequence requirements for this modification. Specifically, NetAcet uses a neural network, which is a *black box* model, for constructing the predictor. Therefore it is difficult to infer the sequence requirements. Similarly, although Motifs tree utilizes a decision tree algorithm, which is a *white box* model, it uses physicochemical sequence features extracted from AAindex [18] as input vectors of the learning, thus preventing a straightforward inference of the sequence requirements of *N*-terminal acetylation from purely a sequence context.

A major objective of this study was to identify rules regarding amino acid sequences that determine the occurrence of *N*-terminal acetylation for nascent proteins whose ^i^Met residues have been removed by methionine aminopeptidase. Establishing these rules will allow us to investigate the roles of *N*-terminal acetylation using protein databases, which would be expected to facilitate studies on the roles of this modification. In consideration of the limitations presented by previous assessment strategies, we used a decision tree algorithm incorporating only the sequence context of the *N*-terminus as input vectors to determine rules that link *N*-terminal sequence and acetylation because this approach provides transparent processing pathways. The performance of the developed tool, *N*-Terminal Acetyl Predictor (NT-AcPredictor), was also compared to existing predictors with respect to accuracy to determine its potential utility as a tool to predict the occurrence of *N*-terminal acetylation.

## Methods

### Dataset

UniProt (Swiss-Prot, ver. 201611) [19] was downloaded from its official website (http://www.uniprot.org/), from which *N* ^α^-acetylated and unacetylated sequences lacking the ^i^Met residues and tagged with both an Evidence Codes Ontology (ECO) code of 0000269 (experimental evidence used in manual assertion) and a PubMed ID(s) were collected. We then looked at the individual *N*-terminal 10-residue sequences and removed duplicate sequences from the dataset, resulting in 411 acetylated (positive) sequences and 701 unacetylated (negative) sequence candidates. We did not remove sequence redundancy by sequence homology because there are many sequences in our dataset that share homologous relationships but their acetylation status is different each other. While the validity of the 411 positive sequences is ensured by the ECO code, we noticed that the absence of a tag “acetylated” is not necessarily equal to “unacetylated”. Therefore, randomly extracted negative sequence candidates were further verified whether there are experimental evidence for not being acetylated by reading the original literature(s) linked through the PubMed ID(s), resulting in collecting 400 verified negative sequences. From this dataset, 400 sequences (positive: 200, negative: 200) were randomly selected as the training dataset, and the remaining sequences (positive: 211, negative: 200) were used as the test dataset. The *N*-terminal sequences of all these 811 proteins are provided in Additional file 1.

### Construction of a predictor

In this study, we constructed a predictor based on the decision tree algorithm, classification and regression tree (CART) [19]. For the learning process, we conducted 5-fold cross-validation of a grid search to identify the best parameter for the maximum depth of the tree, changing the parameter by single digit increments from 2 to 10. We encoded amino acids to one-hot vectors with 20 dimensions using a sparse encoding method in accordance with a frequently used method [16, 20]. The sparse encoding method allowed us to readily infer the biological meanings of the machine learning by connecting a topology of the resultant tree with amino acids on each leaf.

### Performance evaluation metrics

To evaluate the performance of predictors, true positive rate (TPR), specificity (SPC), positive prediction value (PPV), accuracy (ACC), Matthews correlation coefficient (MCC), and F1 score were used. These performance indicators were calculated using the formulas given below, where TP, TN, FP and FN are true positive, true negative, false positive and false negative, respectively.

$$ \begin{array}{c}\mathrm{TPR}=\frac{TP}{TP+ FN}\\ {}\mathrm{SPC}=\frac{TN}{TN+ FP}\\ {}\mathrm{PPV}=\frac{TP}{TP+ FP}\\ {}\mathrm{ACC}=\frac{TP+ TN}{TP+ FP+ FN+ TN}\\ {}\mathrm{MCC}=\frac{TP\times TN- FP\times FN}{\sqrt{\left( TP+ FP\right)\left( TP+ FN\right)\left( TN+ FP\right)\left( TN+ FN\right)}}\\ {}\mathrm{F}1=\frac{2 TP}{2 TP+ FP+ FN}\end{array} $$

## Results

### The first five residues determine the occurrence of *N*-terminal acetylation

**Fig. 2:**
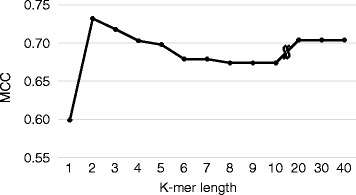
Performance of predictors constructed using various *k*-mer lengths on the training dataset. The *k*-mer length does not include ^i^Met

We first investigated how the *k*-mer length affects the performance of predicting *N*-terminal acetylation. We constructed a variety of predictors by changing *k*-mer length singly from 1 to 10-mers and in 10 steps from 10 to 40-mers, and then evaluated their respective performance on the training dataset using the Mathews correlation coefficient (MCC), which is one of the most robust measures for performance evaluation. As shown in Fig. 2, the MCC value jumped from 1-mer to 2-mer and reached a plateau at 4-mer, suggesting that main sequence determinants of *N*-terminal acetylation for proteins without ^i^Met are located within the *N*-terminal-most 5 residues, with the first two residues being the most important. Also, when we used other criteria such as accuracy or F1 score, the results remained essentially the same. To further investigate whether the important residues are within the *N*-terminal region, we constructed predictors wherein we changed the starting position of the 5-mer input one residue at a time from the *N*-terminus and evaluated the performance of each predictor (Additional file 2: Figure S1). As expected, the best performance was obtained from the predictor constructed using the first five residues. Thus, these results indicate that the amino acid residues that function most strongly in determining the *N*-terminal acetylation reside within the *N*-terminal-most five residues.

### The first position Ser and Ala are the primary determinants of *N*-terminal acetylation

**Fig. 3:**
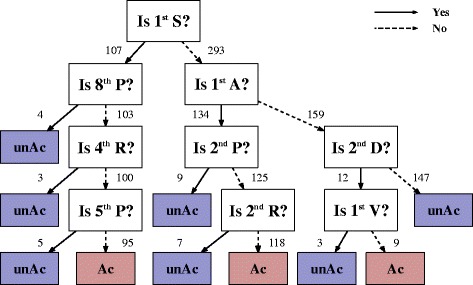
Flowchart of the present predictor, NT-AcPredictor. The decision tree was generated by training the CART algorithm on the training dataset (see “Methods”). The residue numbers in the flowchart do not include ^i^Met. Straight lines and dashed lines with arrows denote “Yes” and “No” paths, respectively. Ac and unAc indicate *N* ^α^-acetylated and unacetylated status, respectively. The numbers shown along with the arrows indicate the number of cases that followed the path. The numbers represent outputs of the learning rather than parameters of the predictor. These results were obtained using the training dataset presented in Additional file 1

As up to 8-mers of identical sequences were contained in the positive and negative datasets, we utilized 10-mers of sequence as input vectors and positive and negative flags as a learning target value for constructing a predictor based on the CART. The resultant flowchart of the decision tree and regular expression of the derived sequence are shown in Fig. 3 and Additional file 2: Figure S2, respectively. As can be seen in the flowchart, the 1st position Ser and Ala were the primary discriminators for the occurrence of *N*-terminal acetylation. The result seems reasonable because the two amino acids are the two most frequent 1st position amino acids of *N*-terminally acetylated proteins in our dataset (Table 1), totaling 87.3% (Ser: 44.0%, Ala: 43.3%) of the acetylated proteins. However, even though the large majority of *N*-acetylated sequences begin with Ser or Ala, these two residues are clearly not an ultimate discriminator for *N*-terminal acetylation as they are also common 1st position residues among the unacetylated proteins (Table 1).

**Table 1:** Residue rankings appearing in the first ten positions of *N*-terminally acetylated and unacetylated proteins

Rank	Sequence position									
	1	2	3	4	5	6	7	8	9	10
Acetylated										
1	S (44.0)	D (17.3)	T (10.9)	A (13.6)	A (12.9)	K (9.7)	A (11.2)	A (14.8)	A (12.7)	A (12.9)
2	A (43.3)	E (14.1)	A (9.0)	G (9.5)	K (9.5)	L (9.5)	K (10.5)	K (9.2)	K (9.7)	G (9.2)
3	T (5.4)	S (11.2)	K (9.0)	E (9.0)	T (9.2)	A (8.8)	V (8.8)	E (8.8)	E (7.5)	L (9.0)
4	G (4.4)	A (10.5)	P (8.5)	V (8.5)	V (8.0)	V (8.3)	S (8.5)	V (8.8)	G (7.3)	K (7.3)
5	V (1.5)	G (7.8)	S (8.5)	S (7.8)	S (7.3)	G (8.0)	L (7.1)	L (8.0)	I (7.1)	E (6.8)
Unacetylated										
1	G (24.3)	K (13.3)	S (16.3)	K (13.8)	S (11.5)	K (10.3)	K (11.0)	K (10.8)	K (10.8)	V (9.8)
2	A (21.5)	L (12.8)	K (9.3)	L (8.8)	A (11.3)	L (9.5)	L (8.5)	R (9.5)	L (10.5)	A (9.3)
3	P (20.8)	R (9.0)	E (6.8)	T (7.8)	K (8.8)	A (8.3)	E (8.5)	S (9.3)	R (8.5)	G (7.8)
4	V (18.8)	A (8.5)	A (6.3)	A (7.3)	D (7.5)	E (8.0)	A (8.3)	E (7.3)	G (8.3)	S (7.8)
5	S (7.8)	P (7.0)	G (6.0)	R (7.0)	V (7.0)	T (7.3)	G (7.5)	V (6.8)	A (7.8)	L (7.3)

Data were taken from the dataset in Additional file 1. The numbers in parentheses represent the percentage frequency of the corresponding amino acid appearance in the respective positions. Only residues ranked within the top five in each position are presented

### The second position constitutes the important discriminator for the occurrence of *N*-terminal acetylation

The trained decision tree revealed that the 2nd position amino acid plays a key role in determining the occurrence of *N*-terminal acetylation (Fig. 3 and Additional file 2: Figure S2). As can be seen in the flowchart, *N*-terminal acetylation occurs when the 1st position is Ala and the 2nd position is not Pro or Arg (A[^PR]), determining 29.5% (=118/400) of the total acetylation states. While *N*-terminal acetylation does not occur when the 1st position is neither Ser nor Ala and the 2nd position is not Asp ([^AS][^D]), determining 36.8% (=147/400) of the total acetylation states. These results indicate that *N*-terminal acetylation is facilitated when Asp is in the 2nd position, while it is inhibited when Pro and Arg are located in the 2nd position. Also, the flowchart shows that *N*-terminal acetylation is facilitated when the 1st position is Ser and the 4th, 5th, and 8th position are not occupied by Arg, Pro and Pro, respectively (SXX[^R][^P]XX[^P]), indicating that 4th position Arg, 5th position Pro, and 8th position Pro are inhibitory to *N*-terminal acetylation. This sequence motif determines 23.8% (=95/400) of the total acetylation state.

To verify and facilitate the interpretation of the results from the predictor output, we examined the residue composition in the first ten positions of *N*-terminally acetylated and unacetylated proteins in our dataset (Table 1). As expected, the 2nd position, the key discriminator for the occurrence of *N*-terminal acetylation suggested by the predictor, was most frequently occupied by one of the two acidic residues, Asp or Glu, in the *N*-terminally acetylated proteins. The frequent appearance of the 2nd position Asp has previously been noted in preceding studies [14, 15]. In contrast, the same position was frequently occupied by one of the two basic amino acids, Arg or Lys in the unacetylated proteins. This finding suggests that the substrate binding site in Nat enzymes that recognizes the 2nd residue prefers acidic residues but excludes basic residues. The X-ray crystal structure of yeast NatA complexed with a substrate has been reported (PDB accession number: 4KVM) [21]. Notably, the substrate binding site of NatA that interacts with the 2nd position of substrates contains two His residues (His 72 and 111). Although, the side-chain of the 2nd position Ala of the substrate that was co-crystalized with NatA does not interact directly with these His residues, these residues may facilitate the interaction with the negatively-charged carboxyl groups of Asp and Glu when the 2nd position of the substrate is Asp or Glu, assuming that the p*K*_a_s of these His imidazole groups are higher than the physiological pH and therefore well protonated at the physiological pH. The hypothesis is supported by the fact that His72 and His111 are 96.7 and 94.7% conserved, respectively, among NatA enzymes from 209 different species (Additional file 2: Table S1), suggesting that the two His residues may play an important role in the catalysis of NatA enzymes. Lastly, although there are two Nat enzymes, NatA and NatD, that act on proteins lacking ^i^Met, it is reasonable to assume that the suggested substrate preference is for NatA because NatD only catalyzes histone H2A and H4 and the 2nd position of these histones in our whole dataset (10 histone H2As and one H4) are occupied by Ser.

### The electrostatic property of the nascent polypeptide chain represents an important determinant of *N*-terminal acetylation

We also noted in the residue rankings of unacetylated proteins that the basic residues Lys and Arg are highly ranked, occurring frequently in the first 10 positions, compared to the acidic residues Asp and Glu (Table 1). The overrepresentation of basic residues in the *N*-terminal region of unacetylated proteins has also been found previously by Polevoda and Sherman [14]. Conversely, it appeared that acidic residues are repeatedly ranked high in the first 10 positions of acetylated proteins (Table 1). To verify the observation, we calculated the charge states of the *N*-terminal 10 residues of acetylated and unacetylated proteins across the whole dataset. In the calculation, we considered only Lys, Arg, Asp, and Glu residues because they are the only residues that have positive or negative charges at physiological pH, and defined their charges to be +1, +1, −1, and −1, respectively. The obtained mean charge states for acetylated and unacetylated proteins were −0.28 (SD = 1.65) and +0.61 (SD = 1.93), respectively, and the difference was statistically significant (*p*-value = 2.0 × 10^−10^) by the Wilcoxon rank-sum test. These results demonstrate that the *N*-termini of acetylated proteins are commonly negatively charged at physiological pH, whereas the *N*-termini of unacetylated proteins are positively charged, suggesting that the electrostatic property of the nascent polypeptide chain comprises an important determinant of *N*-terminal acetylation.

### NT-AcPredictor accurately predicts the occurrence of *N*-terminal acetylation

Finally, we compared our predictor, NT-AcPredictor, with the freely available existing predictors, NetAcet and Motifs tree, using our test dataset. As shown in Table 2, the performance of NT-AcPredictor judged by various measures was superior to that of NetAcet and comparable but slightly worse than that of Motifs tree, demonstrating the comparable predictability of NT-AcPredictor to the best existing predictor. In addition to this benchmark test, we verified the robustness of our algorithm by constructing 10 predictors, each time the training dataset and test dataset was randomly selected by the same manner described in the methods section. The results are shown in Additional file 2: Table S2. The coefficient of variation (CV) for each evaluation criterion was small, thus demonstrating that the effect of random sampling of dataset on the prediction performance is negligible. All the performance indicators of NT-AcPredictor shown in Table 2 were within mean ± SD obtained from the 10 predictors, also demonstrating the robustness of our algorithm.

**Table 2:** Performance comparison of NT-AcPredictor with other predictors

	TPR	SPC	PPV	ACC	MCC	F1
NT-AcPredictor	0.858	0.815	0.830	0.837	0.674	0.844
NetAcet	0.384	0.947	0.929	0.587	0.361	0.544
Motifs tree	0.929	0.845	0.863	0.888	0.778	0.895

TPR, SPC, PPV, ACC, MCC, and F1 represent true positive rate, specificity, positive prediction value, accuracy, Matthews correlation coefficient, and F1 score, respectively. Note that NetAcet was unable to output prediction result for 73 proteins because the predictor did not output the results when the input sequences did not include Ala, Gly, Ser, or Thr at the position from 2 to 4

It is possible that other machine learning methods provide better prediction performance. To explore the possibility, we constructed predictors using random forest and support vector classification (SVC) methods by feeding the same training dataset used for the construction of NT-AcPredictor and evaluated their performances on the same test dataset. The random forest method performed worse and SVC performed slightly better than NT-AcPredictor on most of the performance indicators (data not shown). The reason that random forest could not outperform the decision tree approach might have been the negative influence brought by the probabilistic property of random forest.

## Discussion

Our comparison test showed that the performance of Motifs tree is slightly better than NT-AcPredictor. Even so, the value of using NT-AcPredictor is its unique feature to provide transparent processing pathways from which the sequence determinants of protein *N*-terminal acetylation can be understood. While Motifs tree uses physicochemical sequence features as input vectors rather than just amino acid sequences [17]. Therefore it is difficult to extract the sequence determinants afterward. Since there is a trade-off on the relationship between the prediction performance and perspicuity, this result is understandable. In the performance comparison test, there were 22 cases where NT-AcPredictor outputted correct answers but not Motifs tree, and there were 43 converse cases where Motifs tree outputted correct answers but not NT-AcPredictor. Thus it would be beneficial for users to use both methods in a complementary manner. NT-AcPredictor is available from https://github.com/yamada-kd/nTAcPredictor [22].

When we initiated this study, we hoped to identify clear rules to determine the occurrence of *N*-terminal acetylation for proteins lacking ^i^Met. However, we found it difficult to fully predict the acetylated and unacetylated sequences, suggesting that the substrate specificity of NatA is broad and that there are multiple position-specific preferred and inhibitory residues within the first ten residues, the combinations of which determine the degree of acetylation. However, the number of possible combinations is large, and it is probable that additional position-specific preferred and inhibitory residues remain to be identified. Therefore, these need to be identified to improve the efficacy of our predictor along with a better understanding how their different combinations impact the occurrence of this modification. Other reasons for incomplete predictability may include 1) the substrate specificity of NatA not being the same across species; 2) our whole dataset containing a significant amount of false data; 3) the action of unknown Nat enzymes on the proteins in our whole dataset; and 4) other biological factors influencing this modification other than *N*-terminal sequences. Further studies will be required to better understand the complete determinants of *N*-terminal acetylation.

## Conclusions

We employed a decision tree algorithm to understand rules that linked sequence and *N*-terminal acetylation. Our approach successfully provided several sequence determinants of *N*-terminal acetylation for proteins lacking ^i^Met, demonstrating the usefulness of decision tree-based approaches for studying the sequence determinants of this phenomenon. Although the majority of these sequence determinants have been described previously, novel findings include the facilitating effect of the 2nd position Asp and the inhibitory effect of the 2nd position Pro and Arg on the *N*-terminal acetylation, suggesting that the importance of the 2nd position residue as the key determinant for *N*-terminal acetylation. The developed predictor, NT-AcPredictor, was demonstrated to be able to predict accurately the *N*-terminal acetylation status of proteins for which the *N*-termini had not been experimentally characterized, and thus may be useful to investigate the functional roles of this modification.

## Additional files

Additional file 1: The dataset used in this study. (XLS 101 kb)

Additional file 2: Figure S1. Predictor performance with 5-mer input. **Figure S2**. Regular expression of 10 leaves of the decision tree diagram. **Table S1**. Conservation of His72 and His111 among NatA enzymes. **Table S2**. The mean performance from 10 predictors constructed with randomly selected training dataset. (PDF 262 kb)

## Abbreviations

CART: Classification and regression tree
ECO: Evidence codes ontology
^i^Met: Initiator methionine
MCC: Mathews correlation coefficient
Nat: *N*-terminal acetyltransferases
