# Supplementary material for: Identification of the sequence determinants of protein N-terminal acetylation through a decision tree approach
Source: BMC Bioinformatics. 2017 Jun 2;18:289. doi: 10.1186/s12859-017-1699-4 (PMC5457594; doi:10.1186/s12859-017-1699-4)
Supplement: Supplementary file 2 — Predictor performance with 5-mer input. Figure S2. Regular expression of 10 leaves of the decision tree diagram. Table S1. Conservation of His72 and His111 among NatA enzymes. Table S2. The mean performance from 10 predictors constructed with randomly selected training dataset. (PDF 262 kb) [file 12859_2017_1699_MOESM2_ESM.pdf]

Additional file 2 for "Identification of the sequence determinants of protein *N*-terminal acetylation through a decision tree approach"

Kazunori D. Yamada[1,2*], Satoshi Omori[1], Hafumi Nishi[1] and Masaru Miyagi[3,4]

[1]Graduate School of Information Sciences, Tohoku University, Sendai, 980-8579, Japan. [2]Artificial Intelligence Research Center, National Institute of Advanced Industrial Science and Technology (AIST), Tokyo 135-0064, Japan. [3]Center for Proteomics and Bioinformatics, [4]Department of Nutrition, Case Western Reserve University, Cleveland, OH 44106, USA.

Figure S1

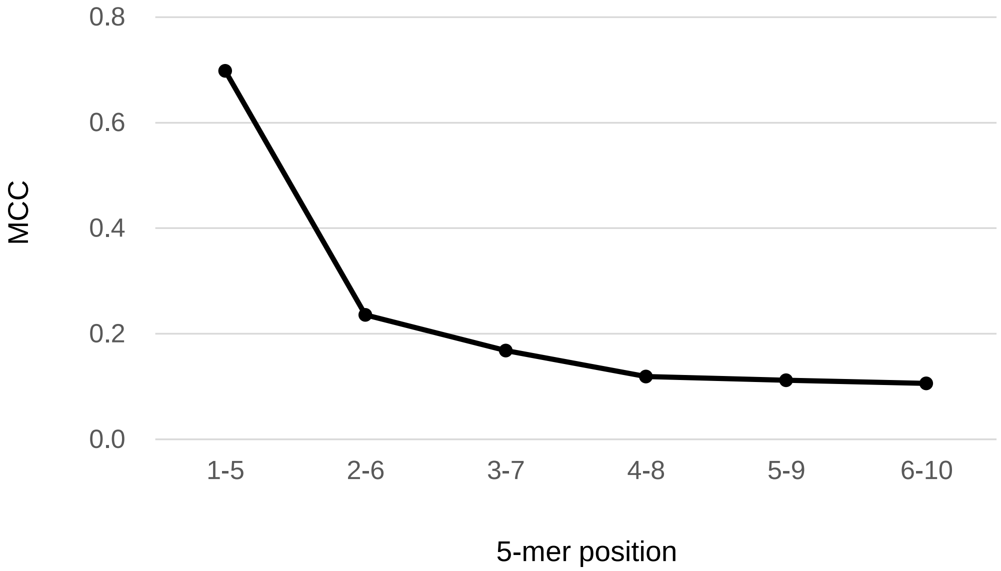

Figure S1. Performance of predictors built using 5-mer input vectors constructed by sliding the 5-mer on various position starting at the 1st residue position excluding ^{i}Met. MCC, Mathews correlation coefficient.

Figure S2

A

0	1	2	3	4	5	6	7	8	9	10
M	S	.	.	[^R]	[^P]	.	.	[^P]	.	.
M	A	[^PR]	.	.	.	.	.	.	.	.
M	[^ASV]	D	.	.	.	.	.	.	.	.

B

0	1	2	3	4	5	6	7	8	9	10
M	S	.	.	.	.	.	.	P	.	.
M	S	.	.	R	.	.	.	[^P]	.	.
M	S	.	.	[^R]	P	.	.	[^P]	.	.
M	A	P	.	.	.	.	.	.	.	.
M	A	R	.	.	.	.	.	.	.	.
M	V	D	.	.	.	.	.	.	.	.
M	[^AS]	[^D]	.	.	.	.	.	.	.	.

Figure S2. Regular expression of 10 leaves of the decision tree diagram. (A) and (B), which include positive and negative classifications for N^{α}-acetylation, respectively. In the figure, the numbers above the vertical lines represent the position of the amino acid. The symbol hat "^" in square brackets indicates the inclusion of any amino acid other than the amino acid(s) shown by the single letter code(s) in the brackets. The symbol dot "." denotes that the position can be occupied by any standard amino acid.

Table S1. Conservation of His72 and His111 among NatA enzymes

Amino acid	Position 72	Position 111
A	0	0
R	0	0
N	0	0.005
D	0	0
C	0	0
Q	0	0
E	0	0
G	0	0
H	0.967	0.947
I	0	0
L	0	0
K	0	0
M	0	0
F	0	0
P	0	0
S	0	0
T	0	0
W	0	0
Y	0	0
V	0	0
-	0.033	0.048

Amino acids located in the positions corresponding to His72 and His111 of human NatA in 209 NatA enzymes from various species are shown in percentage. Here, "-" denotes for deletion.

Table S2. The mean performance of 10 predictors constructed each time with randomly selected training dataset

	TPR	SPC	PPV	ACC	MCC	F1
Mean	0.874	0.778	0.807	0.827	0.657	0.838
SD	0.037	0.039	0.025	0.018	0.036	0.018
CV	0.043	0.050	0.030	0.022	0.054	0.022

TPR, SPC, PPV, ACC, MCC, and F1 represent true positive rate, specificity, positive prediction value, accuracy, Matthews correlation coefficient, and F1 score, respectively. SD and CV stands for standard deviation and coefficient of variation, respectively.
